# Esophageal Atresia: Nutritional Status and Energy Metabolism to Maximize Growth Outcome

**DOI:** 10.3390/children7110228

**Published:** 2020-11-14

**Authors:** Gloria Pelizzo, Francesca Destro, Giorgio Giuseppe Orlando Selvaggio, Luciano Maestri, Margherita Roveri, Alessandra Bosetti, Barbara Borsani, Erica Pendezza, Milena Meroni, Andrea Pansini, Enrico La Pergola, Giovanna Riccipetitoni, Annalisa De Silvestri, Hellas Cena, Valeria Calcaterra

**Affiliations:** 1 Pediatric Surgery Department, “V. Buzzi” Children’s Hospital, 20157 Milano, Italy; francesca_destro@hotmail.com (F.D.); giorgio.selvaggio@asst-fbf-sacco.it (G.G.O.S.); luciano.maestri@asst-fbf-sacco.it (L.M.); margherita.roveri@studenti.unimi.it (M.R.); milena.meroni@asst-fbf-sacco.it (M.M.); andrea.pansini@asst-fbf-sacco.it (A.P.); enrico.lapergola@asst-fbf-sacco.it (E.L.P.); 2 Department of Biomedical and Clinical Science “L. Sacco”, University of Milano, 20157 Milano, Italy; 3 Pediatric Department, “V. Buzzi” Children’s Hospital, 20157 Milano, Italy; alessandra.bosetti@asst-fbf-sacco.it (A.B.); barbara.borsani@unimi.it (B.B.); erica.pendezza@asst-fbf-sacco.it (E.P.); valeria.calcaterra@unipv.it (V.C.); 4 Pediatric Surgery Unit, Fondazione IRCCS Policlinico S. Matteo, 27100 Pavia, Italy; g.riccipetitoni@smatteo.pv.it; 5 Clinical Epidemiology & Biometry, IRCCS Policlinico San Matteo Foundation, 27100 Pavia, Italy; a.desilvestri@smatteo.pv.it; 6 Laboratory of Dietetics and Clinical Nutrition, Department of Public Health, Experimental and Forensic Medicine, University of Pavia, 27100 Pavia, Italy; hellas.cena@unipv.it; 7 Clinical Nutrition and Dietetics Service, Unit of Internal Medicine and Endocrinology, ICS Maugeri IRCCS, 27100 Pavia, Italy; 8 Pediatric and Adolescent Unit. Department of Internal Medicine, University of Pavia, 27100 Pavia, Italy

**Keywords:** esophageal atresia, malnutrition, growth, nutritional status, energy metabolism, feeding difficulty, dysphagia

## Abstract

Background: Long-term negative sequelae of esophageal atresia (EA) may induce poor growth and impaired nutritional status in childhood. We describe the nutritional profile and energy metabolism of children with repaired EA to identify malnutrition risk factors and optimize growth management. Methods: Twenty-one children (>4 years) were included, and anthropometric measurements, nutritional assessment, and energy metabolism were considered. The subjects were defined as undernourished if they met BMI < −2 standard deviation (SD). To grade undernutrition, we defined the prevalence of underweight, stunting, and wasting (cut-off level of <−2 SD). Medical records were reviewed for the type of EA and surgery and perinatal data. Results: Malnutrition was detected in 28.6% of children. Underweight was detected in 23.8% of patients (all with undernutrition *p* < 0.01). Wasting was noted in 28.6% of patients, of these 5 children were undernourished (*p* < 0.001) and stunting was noticed in only one patient with malnutrition (*p* = 0.5). Resting expenditure energy (REE) was lower in undernourished subjects compared to subjects with adequate nutritional status (*p* < 0.001). Malnutrition was associated to: type of EA (*p* = 0.003, particularly type A and C); intervention including deferred anastomosis due to long-gap repair (*p* = 0.04) with/or without jejunostomy (*p* = 0.02), gastric pull-up (*p* = 0.04), primary anastomosis (*p* = 0.04), pyloromyotomy in long-gap (*p* < 0.01); small for gestational age condition (*p* = 0.001). Conclusions: undernutrition risk factors, beyond the type of malformation, surgery, and perinatal factors, must be early considered to personalize nutritional programming. Energy metabolism is important to monitor the nutritional requirements. The management of nutritional issues is surely a contributory factor able to counteract the poor growth of children with EA.

## 1. Introduction

Esophageal atresia (EA) is a rare congenital malformation affecting 1 in 4000 live births [1,2,3]. According to Gross Classification [4], five types of EA have been described, based on the presence and location of the tracheoesophageal fistula (TEF), including EA without TEF (Type A), EA with proximal TEF (Type B), EA with distal TEF (Type C), EA with proximal and distal TEF (Type D), and TEF without EA (Type E). Associated anomalies and syndromes are present in over 50% of children, including vertebral anomalies, anal atresia, cardiac anomalies, tracheoesophageal fistula, renal anomalies, and limb defects (VACTERL) [3,5,6,7].

EA surgical repair and postoperative life support care have led to survival rates up to 90% [8]. Th negative sequelae of EA include feeding difficulties in childhood, long-term dysphagia, and gastrointestinal problems that may have consequences for nutritional status and growth, as well as potential negative outcomes later in life [9,10,11]. A high prevalence of malnutrition, specifically undernutrition, has been reported in children with EA [10,11]. Malnutrition, in general, is the best-studied contributor to poor growth in children [12,13], negatively impacting metabolic profile, immune function, and cardiovascular and respiratory systems. Despite the evidence showing that the nutritional assessment of children should be included as part of the regular clinical practice in EA [14], literature addressing nutritional screening in these children is limited.

The aim of this study was to describe the nutritional status and energy metabolism of EA children. A better understanding of these aspects will lead to the maximization of growth outcomes. Moreover, the correct management of nutritional issues could allow for the prevention of poor growth.

## 2. Materials and Methods

### 2.1. Patients

All patients (*n* = 53) treated for EA between January 2007 and January 2018 at a single institution were offered a nutritional care follow-up. Twenty-one of those have joined the long-term monitoring and were prospectively considered.

In all patients, anthropometric measurements and nutritional assessment were performed. Medical records were reviewed for the type of EA according to the Gross classification [4], demographic and perinatal data, associated anomalies, surgical complications, and esophageal dilatations for stenosis.

The study was performed according to the Declaration of Helsinki and with the approval of the Institutional Review Board. After having received information about the nature of the study, the patient’s parents or tutors gave written consent for their child’s participation.

### 2.2. Anthropometric Measurements

Weight, height, waist circumference (WC), mid-upper arm circumference (MUAC), and skinfold thickness (at the triceps site) were measured.

Body weight was measured to the nearest 100 g with a beam scale, and body height to the nearest 0.1 cm using a vertical stadiometer. BMI (body mass index = weight (kg)/height (m^2^) was calculated and used as a screening tool to classify nutritional status. Weight, height, and BMI z scores were considered according to CDC growth charts.

MUAC was measured at the mid-point between the tip of the shoulder and the tip of the elbow (olecranon process and acromion), using a standard measuring tape.

Skinfold thickness was measured using a professional mechanical skinfold caliper (GIMA, Gessate, Milano, Italy) at the triceps site. Skinfold thickness was measured three times, and the mean value was considered and recorded to the nearest 0.1 mm.

### 2.3. Nutritional Assessment

#### 2.3.1. Nutritional Status

According to BMI z score, the subjects were defined as undernourished if they met BMI< −2 SD (Z-Score < 2) [15,16,17,18].

To grade undernutrition, we defined the prevalence of underweight (low weight-for-age), stunting (low height-for-age) as an indicator of long-term nutritional deprivation, and wasting (low weight-for-height) as an indicator of acute undernutrition.

The prevalence of underweight, stunting, and wasting were calculated at the cut-off level of < −2 SD (Z-Score < 2).

#### 2.3.2. Semistructured Interview for Eating Habits Assessment

A semistructured interview with specific questions aimed to investigate the subjects’ daily intake and feeding habits (varied diet, poor appetite, difficult swallow), was intended and designed by the dietitian specifically for this study and tested for reliability on a small sample of children.

#### 2.3.3. Energy Metabolism

Resting energy expenditure (REE) was measured by indirect calorimetry, using an open-circuit ventilated-hood calorimeter (Sensor Medics 29, Anaheim, CA, USA), and compared to the value obtained by two different predictive equations Harris–Benedict and Schofield Equation, also referred to as LARN (Reference dietary Intake of nutrients and energy for the Italian Population), considering age, gender, weight, and height. Respiratory Quotient was obtained by the ratio of CO_2_ produced/O_2_ consumed, to assess feeding adequacy [19].

## 3. Statistical Analysis

All analyses were performed using Stata 16 (StataCorp, College Station, TX, USA). The data were described with the mean, standard deviation (SD), median, and 25th–75th percentiles if continuous and as counts and percent if categorical. Non-parametric correlations between continuous variables were assessed with the Spearman R test. The association of categorical variables was assessed with Fisher’s exact test. For the purpose of this analysis, biomarkers were dichotomized at the local laboratory cut-off for normality. All tests were 2-sided. A *p*-value < 0.05 was considered statistically significant.

## 4. Results

### 4.1. Features of Patients

Of the 21 patients included in the study, 9 were females and 12 males. The mean age at evaluation was 8.51 ± 2.54 years (range 4.6–12.2 years). According to the Gross classification, esophageal atresia type C occurred in 15 patients (71.4%) and among the remaining patients, 4 (19.0%) were type A, 1 (4.8%) type D, and 1 (4.8%) type B.

The malformation was prenatally detected in 1 patient (9.5%). Associated cardiac malformation was recorded in 7 cases (33.3%), renal agenesis in 1 (4.8%), anorectal anomaly in 2 (9.5%), duodenal atresia in 1 (4.8%); VACTERL syndrome was diagnosed in two of these cases and genetic conditions in 2 others. Prematurity (<37 weeks’ gestation) was detected in 10 subjects (47.6%) and birth weight was small for gestational age (<10th centile for gestational age) in 2 patients (9.52%).

Table 1 and Table 2 describe the clinical features of the enrolled patients and risk factors for malnutrition, respectively. 

### 4.2. Nutritional Assessment

At evaluation, malnutrition (BMI <−2 SDS) was detected in 6 patients (28.6%; 3M/3F). No differences in age (*p* = 0.6) or sex (*p* = 0.1) were noted according to nutritional status (Table 1).

Malnutrition was detected in 5/21 patients (23.8%), all with undernutrition (*p* < 0.01). Wasting was noted in 6/21 patients (28.6%), of these, 5 children were undernourished (*p* < 0.001). Stunting was detected in only one patient with malnutrition (*p* = 0.5).

Undernourished patients showed weight z-score (*p* = <0.01), BMI z-score (*p* < 0.0001), MUAC (*p* < 0.001), and triceps skinfold (*p* < 0.001) significantly lower compared to patients with adequate nutritional status for age and gender. No significant difference was detected in height z-score (*p* = 0.1), Table 1.

Accordingly, BMI and REE were shown to be significantly lower in children with malnutrition compared to subjects with adequate nutritional status (*p* = <0.001). When compared to the referral population (LARN), the predicted REE% was significantly lower in malnourished patients compared to normal-weight children, regardless of the formulas used with two or more predictive variables including age, sex, weight, and height (Table 1). Furthermore, the mean respiratory quotient (RQ) was 0.84 ± 0.07 without significant statistical difference in patients with or without malnutrition.

The features of the patients according to nutritional status are reported in Table 1.

### 4.3. Factors Associated with Malnutrition

As reported in Table 2, the presence of malnutrition was significantly associated to the type of EA (*p* = 0.003) particularly with type A and C, and intervention, deferred anastomosis due to long-gap repair (*p* = 0.04) with/or without jejunostomy (*p* = 0.02), gastric mobilization with partial pull-up (*p* = 0.04), and primary anastomosis (*p* = 0.04).

Excluded pyloromyotomy in long-gap (*p* < 0.01), no other surgical procedures, such as dilatations (*p* = 0.7), gastrotomy (*p* = 0.08), fundoplication (*p* = 0.4) were related to malnutrition, Table 2.

Malnutrition was associated with SGA condition (*p* = 0.001).

No anamnestic symptoms, including recurrent respiratory diseases (*p* = 0.8), coexistence of GERD (gastroesophageal reflux disease) (*p* = 0.2), sleep disorders (*p* = 0.6), and dental problems (*p* = 0.2) was likely associated to malnutrition, Table 2.

Varied diet, poor appetite, difficulties in swallowing were reported in 11 (61.1%), 7 (33.3%), and 5 (23.8%) patients respectively, without significant difference according to nutritional status (*p* = 0.6, *p* = 0.3, and *p* = 0.8, respectively), Table 2.

## 5. Discussion

EA is a congenital abnormality of the esophagus caused by incomplete embryonic compartmentalization of the foregut. Surgical repair aims to restore the esophageal interruption. To date, there is no surgical intervention which receive the best results. Among all types of EA, the long-gap type needs the most demanding surgery, and a primary anastomosis is not feasible. Following esophageal repair, the survival of EA patients exceeds 90%, leading to an increased interest in their long-term morbidity and health-related quality of life [8]. In particular, poor growth is a recognized significant long-term consequence of esophageal atresia repair, although the nutritional and energy status of affected children is poorly studied.

We found a high prevalence of malnutrition, specifically undernutrition, in children with EA. Longitudinal studies have suggested that growth impairment prevalence declines with age [20,21,22,23,24], and recent literature has indicated that this may lead to a “catch-up” growth phenomenon [23,24,25], occurring around 8 years of age. Poorer growth in infancy and early childhood may be related to a higher rate of hospitalizations, feeding difficulties, and complications, such as gastroesophageal reflux and strictures [23,24,25,26]. According to the Australian study reported by Menzies [26], the wasting observed among patients in the current study is indicative of a state of acute undernutrition usually as a consequence of insufficient food intake more than the result of long-term nutritional deprivation. Causes of acute malnutrition are either increased energy expenditure (e.g., infections, increased physical activity), decreased nutrient intake (e.g., lack of caloric intake increments with nutritional formulas), or absorption, thus negatively affecting energy balance. Wasting, in turn, impairs the functioning of the immune system and can lead to increased susceptibility to infectious diseases, perpetuating a vicious circle. These results underline the crucial role of a careful nutritional assessment and intervention during development, in order to prevent comorbidity.

In our population, the poor growth also after catch-up growth, not related to gastrointestinal symptoms. We noted that malnutrition is related to the type of EA and consequently to surgical factors. The repair of long-gap EA, achieved through delayed primary anastomosis, seems to be an unfavorable factor; jejunostomy, gastric pull-up, and pyloromyotomy appear to be additional negative prognostic factors. Even though long-gap EA needs the most demanding surgical treatment among all types of EA, in literature, there is no consensus on the definition of long-gap and usually it is described as a “non-feasible primary anastomosis”. Up to date, two different surgical attitudes have been followed: the replacement of the gastric tube (by gastric pull-up, jejunal interposition, gastric tube reconstruction and colon interposition), or the preservation of the native esophagus (by elongation and traction such as Foker or Kimura). Additionally, the replacement technique is described under both retrosternal and mediastinal approach and it is not always associated with an antireflux procedure; in the same way, the different use of right or of left part of the colon was described. The same considerations have been applied for the diverse techniques used for esophageal elongation consisting of Sharli or Collis technique (lower esophageal bud) or modified techniques of stretching (Kimura and Focker technique). No specific evidence of the positive or negative effects of these several surgical techniques on nutritional status has been reported. Therefore, it is difficult to define a causal role between surgery and malnutrition. Abnormalities of absorption related to mucosal inflammation or anatomic abnormalities should also be considered. Our data on negative nutritional impact of jejunostomy also support the role of long-term effects of malabsorption.

In addition, it is not excluded a negative impact of decreased gastric volume in patients with pyloroplasty. Prolonged hospitalization and complications associated with long-gap EA should be carefully addressed since are crucial factors of poor growth in the first years of life.

However, during childhood and adolescence, other causal factors, such as inadequate nutrients and energy intake, can interfere; indeed, while the whole sample has shown to consume a varied diet, this does not necessary correspond to an adequate diet [27] able to fulfill all the nutritional requirements during growth, especially in surgical children at higher risk of malnutrition. In confirmation of this, it is interesting to observe that although RQs were within physiological trends with no significant difference between groups, the mean RQ value of undernourished children was actually lower shifting towards values below 0.85, suggesting the use of endogenous fat stores to meet energy requirements during underfeeding.

In children suffering from undernutrition, we measured a lower REE, as expected, given that they had lower weight-for-age than children with an adequate nutrition state. Moreover, the measurement of REE differed more from the estimated one, obtained from predictive formulas, in the undernourished group than in normally nourished children. These results confirm that it is essential to carry out an Indirect Calorimetry in all children at risk of malnutrition, including surgical care ones, and not to rely on predictive formulas suitable only for the general population.

Our results confirm that the low birth weight in children with EA was significantly associated with poor growth [5,25,28]. However, considering the lack of data on the catch growth of children with SGA, further longitudinal studies using consistent parameters are required to clarify the direct contribution of this factor.

Children with EA transition into adulthood showing multiple problems. Dysphagia and other gastrointestinal complications, such as GERD, feeding difficulties, esophageal strictures, and respiratory issues are negative sequelae of EA and are considered as main causes of nutritional problems and poor growth [9,10,11].

Despite the fact that these symptoms were frequently recorded in our patients, surprisingly, there were no associations between self-reported gastrointestinal symptoms and malnutrition. These data support the hypothetical coexistence of other possible factors.

As reported by Birketvedt, an inadequate intake of energy in children and adolescents with EA should always be considered [29]. This finding underlines the importance of close follow-ups, with a periodical assessment of the nutritional status to ensure optimal growth, including Indirect Calorimetry measurement. REE measurement will help not only to reach accuracy in medical nutritional treatment but also to individualize the nutrition support, especially in those patients with altered body composition, continued weight loss, persistent inflammatory state, and all those who fail to respond to presumed adequate nutrition support [30].

Additionally, poor growth may be related to impaired metabolism or absorption, resulting from an altered intestinal microbiota [30,31]. In these children, the esophageal discontinuity prevented organisms normally acquired in utero or during parturition from colonizing the infant’s gut, resulting in an imbalance in the gut microbial community [31,32]. Additionally, frequent antibiotic use for respiratory infections, and the early and prolonged use of high-dose proton pump inhibitors for GERD, may contribute to dysbiosis in children with EA influencing nutritional status and overall health. Finally, evidence shows that diet and malnourishment affect the physiological development of the gut microbiota in early childhood, leading to an altered composition that lacks the required functions for healthy growth [31].

Considering data from our study and nutritional requirements across infancy, childhood, and puberty, we suggest that key indexes of nutritional status should be monitored at least three time a year in the first 2 years of life and during puberty and twice a year in patients aging from 2 to 9 years of age, in order to assess that the energy intakes of children are adequate to support satisfactory growth.

We acknowledge some study limitations, including the relatively small sample size and the wide age range of patients. A larger sample size is mandatory to perform a predictive model using logistic regression in order to define the association between malnutrition and risk factors.

We considered all AE subtypes together and the differences between subtypes are not excluded; however, all AE are similar in repeated and prolonged hospitalization, multiple procedures, and high malnutrition risk related to feeding problems.

In addition, the long period used to collect the clinical data could be considered as a confounding factor; however, there was not any significant change in the surgical technique and a senior and expert surgeon was always present during the operation.

A longitudinal study is desirable to define the role of time and growth in the development of malnutrition. A semi-structured interview on nutritional intake was also performed and the potential recall bias was not excluded. Finally, we recommend recording physical activity in future studies, as it is an important contributor of energy expenditure. The lack of biochemical data may also represent a diagnostic limit for a precise malnutrition classification, especially for the assessment of micronutrient deficiencies.

Despite the acknowledged limitations, this is one of the few studies in the scientific literature evaluating malnutrition in children with EA and considering resting energy expenditure and energy requirements; it may be considered a first step suggesting the necessity of multidisciplinary investigation on children with EA, in order to expand our knowledge of malnutrition risk factors, identifying early interventions aimed at improving health status and preventing complications. A long-term observation/intervention study will be useful to optimize our results.

## 6. Conclusions

In conclusion, we confirm that children with EA are particularly at risk of poor nutrition. The risk factors for poor growth, including the type of malformation, surgery, perinatal factors, must be considered as soon as possible to tailor a personalized nutritional program according to age and individual need. A strict auxological monitoring is required in infants. In younger children and adolescents, with a collaborative attitude, the measurement of resting energy expenditure represents an important tool to assess adequacy of nutritional intake. A multidisciplinary specialist team for the evaluation and treatment of children with EA is recommended, assessing the adequacy of nutritional support, monitoring improvements over time, and defining preventive strategies to limit malnutrition and long-term consequences.

## Figures and Tables

**Table 1:** Clinical features and energy metabolism of the patients according to nutritional status.

	Total (*n* = 21)	Adequate Nutritional Status (*n* = 15)	Undernutrition (*n* = 6)	*p*
Age (yrs)	8.51 (2.34)	9.03 (2.34)	7.2 (1.92)	0.10
Sex (M/F)	12/9	9/6	3/3	0.67
Weight kg	24.30 (7.31)	27.23 (5.99)	16.9 (4.67)	<0.001
Weight z-score	−0.88 (−1.62–−0.5)	−0.65 (−1.3–0.01)	−2.73 (−3.21—2.18)	<0.001
Height cm	127 (14.34)	132 (11.33)	115 (15.01)	0.01
Height z-score	−0.55 (−1.36–0.41)	−0.28 (−1.09–0.7)	−1.525 (−1.96–−0.76)	0.10
BMI kg/m^2^	14.68 (1.85)	15.51 (1.48)	12.6 (0.62)	<0.001
BMI z-score	−1.33 (1.41)	−0.64 (0.94)	−3.05 (0.7)	<0.001
Height-for-age (stunting) z-score	−0.1 (−1.1–0.5)	−0.1 (−0.7–1.6)	−1.2 (−1.9–0.1)	0.14
Weight-for-height (wasting) z-score	1.6 (−2–2.5)	2 (0.4–2.9)	−2.9 (−3.2—2.5)	<0.001
MUAC cm	17.78 (2.48)	18.91 (1.81)	14.97 (1.42)	<0.001
Waist circumference cm	55.33 (6.12)	58.07 (4.87)	48.5 (2.19)	<0.001
Triceps skinfold mm	8.56 (2.97)	9.28 (3.19)	6.77 (1.13)	0.07
VO_2_ L/min	0.15 (0.02)	0.16 (0.01)	0.13 (0.01)	<0.001
VCO_2_ mL/min	0.13 (0.02)	0.13 (0.02)	0.11 (0.01)	0.01
RQ	0.84 (0.07)	0.85 (0.09)	0.82 (0.01)	0.44
REE kcal/d	1035 (142.64)	1098.93 (101.23)	856 (62.42)	<0.001
REE kcal/kg/d	43.70 (8.06)	50.49 (8.09)	41.28 (6.76)	0.02
Theoretical Basal Metabolic Rate kcal/d	1037.16 (127.04)	1081.07 (89.78)	914.2 (144.16)	<0.01
Theoretical Basal Metabolic Rate kcal/kg/d	43.95 (9.04)	53.93 (8.3)	40.58 (6.75)	<0.01
LARN REE ^1^ kcal/d	1153.68 (174.01)	1180 (184.14)	1080 (129.81)	0.28
Schofield formula weight ^2^ kcal/d	1053.16 (163.33)	1120.22 (131.18)	885.5 (106.29)	<0.001
Schofield formula weight and height ^3^ cal/d	1096.5 (908.72–1173)	1157 (1042.7–1210.6)	843.03 (813.42–876)	<0.001

^1^ Reference Intake of nutrients and energy for Italian Population for age and sex (LARN), ^2^ Schofield Formula for age, sex and weight applied to the Reference Intake of nutrients and energy for Italian Population (LARN), ^3^ Schofield Formula for age, sex, weight, and height applied to the Italian benchmarks of nutrient intake levels (LARN).

**Table 2:** Risk factors for malnutrition.

Recorded Features	Total (*n* = 21)	Adequate Nutritional Status (*n* = 15)	Undernutrition (*n* = 6)	*p*
**Perinatal features**				
Type of AE				
Type A	4 (19.05)	1 (6.67)	3 (50)	0.03
Type B	1 (4.76)	0 (0)	1(16.67)	
Type C	15 (71.4)	13 (86.67)	2 (33.33)	
Type D	1 (4.76)	1 (6.67)	0 (0)	
SGA	2 (9.52)	0 (0)	2 (33.3)	0.01
**Surgical procedures**				
Deferred anastomosis for long gap *	7 (33.33)	3 (20)	4 (66.67)	0.04
Primary anastomosis	14 (66.67)	12 (80)	2 (33.33)	0.04
Gastric mobilization and “limited” pull-up	7 (33.33)	3 (20)	4 (66.67)	0.04
Jejunostomy	4 (19.05)	1 (6.67)	3 (50)	0.02
Pyloromyotomy	3 (14.29)	0 (0)	3 (50)	<0.01
Gastric transposition	1 (4.76)	0 (0)	1 (16.67)	0.10
Gastrostomy	8 (38.1)	4 (26.67)	4 (66.67)	0.08
Dilatations	12 (57.14)	9 (60)	3 (50)	0.67
Fundoplication	6 (28.57)	5 (33.33)	1 (16.67)	0.44
**Gastrointestinal symptoms**				
Varied diet	11 (61.11)	9 (64.29)	2 (50)	0.60
Poor appetite	10 (55.56)	7 (50)	3 (75)	0.37
Swallowing difficulty	5 (27.78)	4 (28.57)	1 (25)	0.88
Retrosternal pain	6 (33.33)	4 (28.57)	2 (50)	0.42
Regurgitations of food	10 (55.56)	8 (57.14)	2 (50)	0.79
Nausea	3 (16.67)	2 (14.29)	1 (25)	0.61
Vomiting during meal	4 (22.22)	3 (21.43)	1 (25)	0.87
Epigastric pain	5 (27.78)	4 (28.57)	1 (25)	0.88
Diarrhea	7 (38.89)	4 (28.57)	3 (75)	0.09
**Other conditions**				
Recurrent respiratory diseases	13 (61.9)	9 (60)	4 (66.67)	0.77
Asthma	8 (38.1)	5 (33.33)	3 (50)	0.47
Sleep disorders	5 (27.78)	4 (28.57)	1 (25)	0.60
Dental problems	4 (22.22)	4 (28.57)	0 (0)	0.22
Voice changes	6 (33.33)	6 (42.86)	0 (0)	0.10
Recurrent acute otitis media	1 (5.56)	1 (7.14)	0 (0)	0.58

* Deferred anastomosis was preceded by gastrostomy at birth in all 7 patients together with prevertebral anchorage of the esophageal stumps in 1 case. Subsequently, the anastomosis was successfully performed in 6/7 patients at a mean age of 4 months [age range 1–8 months] with extended mobilization of the lower esophageal pouch together with the stomach (“limited gastric pull-up”). One patient out of 7, required complete gastric transposition following refractory anastomotic stenosis at 6 months.
